# Financial Difficulties Correlate With Mental Health Among Bangladeshi Residents Amid COVID-19 Pandemic: Findings From a Cross-Sectional Survey

**DOI:** 10.3389/fpsyt.2021.755357

**Published:** 2021-12-08

**Authors:** Abu Bakkar Siddique, Sudipto Deb Nath, Md. Saiful Islam, Tausif Hasan Khan, Shahina Pardhan, M. Ziaul Amin, M. Imran Al Amin, Zayed Bin Zakir Shawon, Kamrun Nahar Koly

**Affiliations:** ^1^Department of Public Health and Informatics, Jahangirnagar University, Dhaka, Bangladesh; ^2^Centre for Advanced Research Excellence in Public Health, Dhaka, Bangladesh; ^3^Army Medical College Jashore, Jashore, Bangladesh; ^4^Department of Chemical Engineering, Bangladesh University of Engineering and Technology, Dhaka, Bangladesh; ^5^Vision and Eye Research Institute, School of Medicine, Anglia Ruskin University, Cambridge, United Kingdom; ^6^Department of Genetic Engineering & Biotechnology, Jashore University of Science & Technology, Jashore, Bangladesh; ^7^Dhaka Medical College Hospital, Dhaka, Bangladesh; ^8^Department of Mathematics and Natural Sciences, Brac University, Dhaka, Bangladesh; ^9^Health System and Population Studies Division, International Centre for Diarrhoeal Disease Research, Dhaka, Bangladesh

**Keywords:** mental health, anxiety, depression, stress, financial difficulties, COVID-19

## Abstract

**Background:** The COVID-19 pandemic is a global threat which has challenged mental resilience and impacted the psychological well-being of people across all age groups globally. The present study aimed to investigate how financial difficulties during the pandemic correlate with mental health among residents of Bangladesh.

**Methods:** A cross-sectional survey was conducted on 4,020 residents from different parts of Bangladesh between July and September 2020, during a period of elevated risk of COVID-19 infection. A self-reported online questionnaire comprising socio-demographic, financial difficulties and psychometric measures (to assess depression, anxiety and stress) was used to gather information from participants. Multivariable logistic regression analysis was performed to determine the factors associated with mental health consequences.

**Results:** The prevalence of depression, anxiety, and stress in the sample were 71.1%, 62.3%, and 56.7%, respectively. Levels of depression, anxiety, and stress were significantly higher among participants who reported female sex, being unmarried, smaller families, higher monthly family income, poor self-perceived health status, living near people who had been infected by COVID-19, probability of decreased income, food scarcity (both during the pandemic and in the future) and the possibility of unemployment. However, due to the nature of the cross-sectional study performed with a convenience sampling method, the causal relationship between variables cannot be justified.

**Conclusions:** After several months of the COVID-19 pandemic in Bangladesh, more than half of the respondents rated their mental health concerns as moderate to severe. The findings highlight the contributing factors of poor mental health which warrant the creation of interventions that address the economic, financial and mental health impacts of the pandemic.

## Introduction

The world is undergoing an uncertain coronavirus disease 2019 (COVID-19) pandemic which has cost millions of lives, impacted the economy, and had severe mental health consequences. Bangladesh reported its first COVID-19 case on March 8, 2020 (1, 2). Since then, the highly contagious virus has spread rapidly with 1,545,800 confirmed cases and 27,277 deaths as of September 22nd 2021 (3). To prevent the spread of the virus, the Bangladesh government imposed a nationwide as well as partial (zonal) lockdown, home quarantine, and travel restrictions, similarly to other countries (4).

Pandemic related issues including fear of infection and losing loved ones, spread of misinformation, lack of medical treatment and shortage of properly equipped units to treat patients; along with lockdown-related stress (i.e., prolonged home isolation, social distancing, food insecurity, fear of unemployment, loss of income, etc.) have been associated with poor mental health. Common mental health issues include depression, anxiety, phobia, insomnia, and trauma (5–13). There is evidence that people may experience symptoms of psychosis, anxiety, trauma, suicidal ideation, and panic over communicable disease outbreaks (14, 15). While the world is combating the physical effects of COVID-19, mental health has often been neglected or left unaddressed (16).

Many of the world's nations, including Bangladesh, declared emergency lockdown measures to reduce transmission of the virus (17). During the COVID-19 pandemic, particularly in lockdown periods, people experienced multiple financial difficulties including but not limited to unemployment, job scarcity, income loss, and food insecurity (18, 19). Such financial difficulties, along with worries related to social distancing, isolation and quarantine may predispose people to common mental health issues such as anxiety, depression, and stress (18, 20). Most literature relating to this pandemic focuses on the genomic characterization of the virus, epidemiology, clinical identification features of infected patients and challenges for global health governance (21–24). In brief, through a global public health emergency such as the one we are currently experiencing, it is important to examine the pandemic's psychological effect on populations to establish symptom mitigation strategies (25).

Multiple studies have reported an increase in anxiety, depression, and stress during the COVID-19 pandemic (4, 13, 26–36). One potential reason could be the pandemic's influence on the economy and workforce. The negative effects of the pandemic on the economy as well as an increase in unemployment may have contributed to the higher levels of anxiety and depression in a unique way (37, 38). A recent study reported that the mental health of Canadian adults declined significantly during the early phase of the pandemic, correlated with economic concerns (39). Unprecedented increases in unemployment, economic uncertainty, and financial concerns are all key factors contributing to the rise in mental health problems during the pandemic (40). A longitudinal study conducted among British students prior to the pandemic revealed that mental health was associated with financial difficulties (41).

In this context, it is very important for us to know the financial issues and factors associated with mental health during the COVID-19 pandemic among residents of lower- and middle-income countries like Bangladesh. We sought to investigate the correlation between financial difficulties and mental health among Bangladeshi residents as there is scarce existing literature on this topic. We hypothesized that financial difficulties (e.g., decreased income and food scarcity, along with the possibility of future decreasing income and food scarcity) would be associated positively with poor mental health. Our study will help the Government and policy makers to resolve financial problems and to expand psycho-social support among the residents of Bangladesh.

## Methods and Materials

### Participants and Procedure

A cross-sectional study was conducted among house-bound residents in Bangladesh from different cities (including Dhaka, Narayangonj, Sylhet, Chittagong, Mymensingh and Cox's Bazar) between July and September 2020. The data were collected utilizing an online survey tool (*Google Forms*) with a convenience sampling design. An online survey was used to avoid the possibility of infection and to maintain spatial distancing. After incorporating all questions in *Google Forms*, a shareable link was generated. The survey link was disseminated via online platforms including Facebook, Messenger, and WhatsApp in order to get a rapid response and cover a geographically diverse area of Bangladesh. Initially, 5057 participants submitted a response. After removing incomplete surveys, 4,020 were kept for final analysis. All surveys were completed in the Bengali language. The inclusion criteria included being (i) able to read Bangla, (ii) aged 18 years or over, and (iii) able to complete the entire survey. All respondents gave virtual informed consent.

### Sample Size Calculation

The sample size was calculated using the following Equation (1):


(1) $$\begin{array}{l} \;n\;=\frac{z^{2}pq}{d^{2}}\;\\ \Rightarrow n\;=\frac{1.96^{2}\;\times \;0.5\;\times \;\left(1-0.5\right)}{0.05^{2}}\;\\ \Rightarrow n\;=384.16\approx 384 \end{array}$$

Here, *n* = number of samples

*z* = 1.96 (95% confidence level)

*p* = prevalence estimate (50% or 0.5)

*q* = (1-*p*)

*d* = precision limit or proportion of sampling error (0.05).

We hypothesized that the prevalence estimate (p) for the present study would be 50%. Assuming a 10% non-response rate, a sample size of 423.5 ≈ 424 participants was estimated. Our sample size exceeded this estimate.

### Measures

A self-reported questionnaire including questions concerning socio-demographics and financial difficulties along with a psychometric scale (i.e., DASS-21—see below) was used to collect data from participants.

#### Socio-Demographics Information

Socio-demographic questions section included those on age, sex, monthly family income, educational qualifications, marital status, size of family, habitat (rural/urban), and self-reported health status (good/poor).

In addition, “yes/no” questions were included in the survey regarding the effects of COVID-19 on the participants' family members and their close networks (neighbors and friends).

#### Measures of Financial Difficulties During the COVID-19 Pandemic

With regard to financial difficulties, participants were asked whether they thought that their income status had changed during the COVID-19 pandemic (yes/no), the probability of decreasing income in the future (yes/no), the possibility of losing their job in future (yes/no), food scarcity during the pandemic (yes/no), the probability of food scarcity in the future (yes/no). The Cronbach's alpha of these measures was 0.78.

#### Depression Anxiety Stress Scale (DASS-21)

Depression, anxiety, and stress were assessed utilizing the DASS-21 (42) comprising 21 items and three dimensions (seven items per dimension) (e.g., “*I could not seem to experience any positive feeling at all*” for depression; “*I was worried about situations in which I might panic*” for anxiety; and “*I found it difficult to relax*” for stress) responded to on a four-point Likert scale from 0 (*Did not apply to me at all*) to 3 (*Applied to me very much*, or *most of the time*). Higher scores on each dimension reflect higher depression, anxiety, and stress, respectively. Scoring of the sub-scales was as follows—depression: normal 0–9, mild 10–13, moderate 14–20, severe 21–27, and extremely severe +28; anxiety: normal 0–7, mild 8–9, moderate 10–14, severe 15–19, and extremely severe +20, and stress: normal 0–14, mild 15–18, moderate 19–25, severe 26–33, and extremely severe +34). In the present study, the Cronbach's alpha for depression, anxiety and stress were very good (0.80, 0.82, and 0.81 respectively) and the total was 0.80.

### Statistical Analysis

Descriptive analysis including frequencies and percentages were performed for categorical variables, while means, standard deviations, etc. were calculated for continuous variables. We present prevalence estimates of depression, anxiety and stress using the predefined thresholds above. All items of the DASS-21 yielded Skewness and Kurtosis values within the ± 2.0 range, indicating that they were normally distributed (43). Chi-square tests were performed to determine the significant relationship of depression, anxiety, and stress with all examined variables. In addition, binary logistic regression analysis was performed to determine the candidates for multivariable logistic regression analysis. The variables that were statistically significant (*p* < 0.05) in the binary logistic regression analysis of depression, anxiety, and stress were included in the multivariable regression models. The multicollinearity was checked using tolerance (>0.1) and variance inflation factor (VIF; <10) before performing multivariable logistic regression. Analyses were performed using Statistical Package for Social Science (SPSS) version 25.0.

### Ethics

The survey was conducted according to the guidelines of the Helsinki Declaration 1975. The study protocol was reviewed and approved by the Ethical Review Committee of the Faculty of Biological Science and Technology, Jessore University of Science & Technology, Jessore-7408, Bangladesh [Ref: ERC/FBS/JUST/2020-43(a)]. All respondents were informed about the purpose of the study, the procedure, and the right to withdraw their data. Each participant gave virtual informed consent prior to completing the study. Participants were informed that all their information would be kept anonymous and confidential, and they were provided with information about the nature and purpose of the study.

## Results

### General Characteristics of Participants

A total of 4,020 participants (male: 53.6%) were included in the final analysis. Most were younger, aged between 18 and 25 years (86%). The majority were educated up to university level (78.9%), unmarried (87.8%), resided in urban areas (76.9%), had up to 4 members in their immediate family (54%) and a family monthly income ranging from 20,000–50,000 Bangladeshi Taka [BDT; 84.87 BDT = 1 US$] (47.6%). 7.3% reported poor self-perceived health status.

Almost 6% reported that COVID-19 had affected their family members, while the majority reported that COVID-19 had affected people in their residential area (72.3%). Over two-thirds of participants reported their income had decreased during the COVID-19 pandemic (66.7%), while 72.7% reported the possibility of decreased income in the future, and 35.3% reported the possibility of losing their job during the pandemic. In addition, 17.2% reported current food scarcity, with almost half of participants thinking food scarcity was possible in the future (45.3%).

### Mental Health Consequences and Their Association With Other Variables

**Figure 1:**
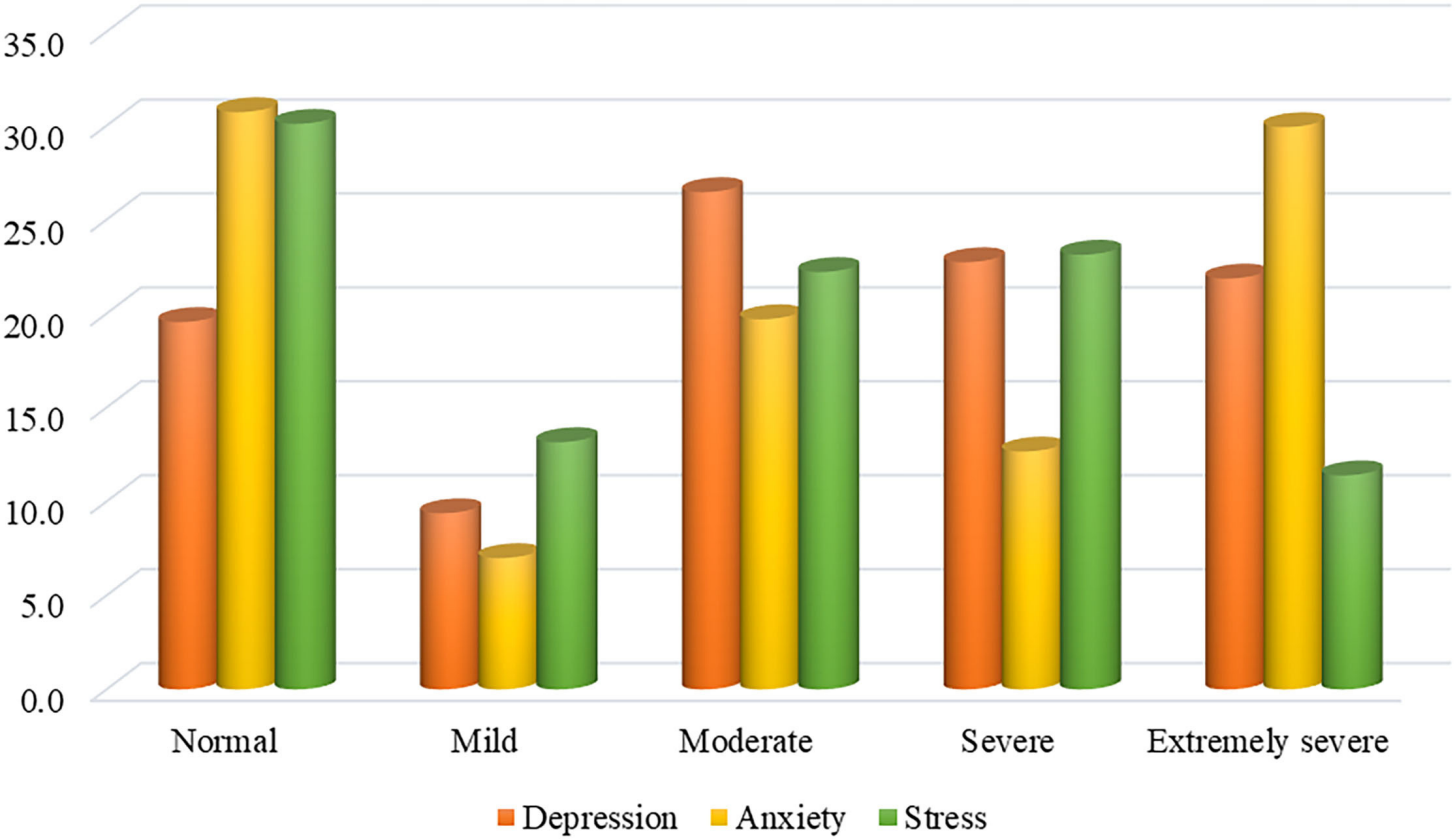
Levels of mental health symptoms.

Figure 1 presents the patterns of mental health concerns among Bangladeshi residents during the COVID-19 pandemic. The prevalence estimates of moderate to severe depression, anxiety, and stress were 71.1, 62.3, and 56.7%, respectively. As per as bivariate analysis (Chi-square tests), most variables were significantly associated with depression, anxiety, and stress (Table 1).

**Table 1:** Distribution of all examined variables and association with depression, anxiety, and stress.

**Variables**	**Overall** ***N*** **= 4,020**	**Depression**			**Anxiety**			**Stress**		
		**Negative**	**Positive**	***P*** **-value**	**Negative**	**Positive**	***P*** **-value**	**Negative**	**Positive**	***P*** **-value**
		***n*** **(%)**	***n*** **(%)**		***n*** **(%)**	***n*** **(%)**		***n*** **(%)**	***n*** **(%)**	
**Age**										
Younger age	3,458 (86)	955 (27.6)	2,503 (72.4)	<0.001	1,318 (38.1)	2,140 (61.9)	0.309	1,440 (41.6)	2,018 (58.4)	<0.001
Middle age	512 (12.7)	192 (37.5)	320 (62.5)		183 (35.7)	329 (64.3)		273 (53.3)	239 (46.7)	
Older age	50 (1.2)	16 (32)	34 (68)		15 (30)	35 (70)		26 (52)	24 (48)	
**Sex**										
Male	2,153 (53.6)	671 (31.2)	1,482 (68.8)	0.001	898 (41.7)	1,255 (58.3)	<0.001	1,064 (49.4)	1,089 (50.6)	<0.001
Female	1,867 (46.4)	492 (26.4)	1,375 (73.6)		618 (33.1)	1,249 (66.9)		675 (36.2)	1,192 (63.8)	
**Education**										
Below university	847 (21.1)	203 (24)	644 (76)	<0.001	247 (29.2)	600 (70.8)	<0.001	338 (39.9)	509 (60.1)	0.029
University	3,173 (78.9)	960 (30.3)	2,213 (69.7)		1,269 (40)	1,904 (60)		1,401 (44.2)	1,772 (55.8)	
**Marital status**										
Married	489 (12.2)	193 (39.5)	296 (60.5)	<0.001	174 (35.6)	315 (64.4)	0.320	267 (54.6)	222 (45.4)	<0.001
Unmarried	3,531 (87.8)	970 (27.5)	2,561 (72.5)		1,342 (38)	2,189 (62)		1,472 (41.7)	2,059 (58.3)	
**Family members**										
Up to 4	2,172 (54)	568 (26.2)	1,604 (73.8)	<0.001	791 (36.4)	1,381 (63.6)	0.145	881 (40.6)	1,291 (59.4)	0.001
5 to 8	1,593 (39.6)	505 (31.7)	1,088 (68.3)		630 (39.5)	963 (60.5)		732 (46)	861 (54)	
Above 8	255 (6.3)	90 (35.3)	165 (64.7)		95 (37.3)	160 (62.7)		126 (49.4)	129 (50.6)	
**Monthly income (BDT)**										
<20,000	712 (17.7)	223 (31.3)	489 (68.7)	0.021	302 (42.4)	410 (57.6)	<0.001	348 (48.9)	364 (51.1)	<0.001
20,000 to 50,000	1,913 (47.6)	514 (26.9)	1,399 (73.1)		641 (33.5)	1,272 (66.5)		774 (40.5)	1,139 (59.5)	
>50,000	1,395 (34.7)	426 (30.5)	969 (69.5)		573 (41.1)	822 (58.9)		617 (44.2)	778 (55.8)	
**Residence**										
Rural	930 (23.1)	282 (30.3)	648 (69.7)	0.286	354 (38.1)	576 (61.9)	0.817	435 (46.8)	495 (53.2)	0.014
Urban	3,090 (76.9)	881 (28.5)	2,209 (71.5)		1,162 (37.6)	1,928 (62.4)		1,304 (42.2)	1,786 (57.8)	
**Self-perceived health status**										
Good	3,728 (92.7)	1,107 (29.7)	2,621 (70.3)	<0.001	1,458 (39.1)	2,270 (60.9)	<0.001	1,657 (44.4)	2,071 (55.6)	<0.001
Poor	292 (7.3)	56 (19.2)	236 (80.8)		58 (19.9)	234 (80.1)		82 (28.1)	210 (71.9)	
**COVID-19 affected people in family**										
Yes	237 (5.9)	60 (25.3)	177 (74.7)	0.206	58 (24.5)	179 (75.5)	<0.001	86 (36.3)	151 (63.7)	0.026
No	3,783 (94.1)	1,103 (29.2)	2,680 (70.8)		1,458 (38.5)	2,325 (61.5)		1,653 (43.7)	2,130 (56.3)	
**COVID-19 affected people in area**										
Yes	2,907 (72.3)	758 (26.1)	2,149 (73.9)	<0.001	1,008 (34.7)	1,899 (65.3)	<0.001	1,158 (39.8)	1,749 (60.2)	<0.001
No	1,113 (27.7)	405 (36.4)	708 (63.6)		508 (45.6)	605 (54.4)		581 (52.2)	532 (47.8)	
**Income status change due to COVID-19**										
Yes	2,683 (66.7)	667 (24.9)	2,016 (75.1)	<0.001	886 (33)	1,797 (67)	<0.001	1,043 (38.9)	1,640 (61.1)	<0.001
No	1,337 (33.3)	496 (37.1)	841 (62.9)		630 (47.1)	707 (52.9)		696 (52.1)	641 (47.9)	
**Possibility of decreasing income**										
Yes	2,923 (72.7)	706 (24.2)	2,217 (75.8)	<0.001	974 (33.3)	1,949 (66.7)	<0.001	1,144 (39.1)	1,779 (60.9)	<0.001
No	1,097 (27.3)	457 (41.7)	640 (58.3)		542 (49.4)	555 (50.6)		595 (54.2)	502 (45.8)	
**Probability of losing job**										
Yes	1,421 (35.3)	267 (18.8)	1,154 (81.2)	<0.001	354 (24.9)	1,067 (75.1)	<0.001	505 (35.5)	916 (64.5)	<0.001
No	2,599 (64.7)	896 (34.5)	1,703 (65.5)		1,162 (44.7)	1,437 (55.3)		1,234 (47.5)	1,365 (52.5)	
**Food scarcity due to COVID-19**										
Yes	692 (17.2)	90 (13)	602 (87)	<0.001	101 (14.6)	591 (85.4)	<0.001	206 (29.8)	486 (70.2)	<0.001
No	3,328 (82.8)	1,073 (32.2)	2,255 (67.8)		1,415 (42.5)	1,913 (57.5)		1,533 (46.1)	1,795 (53.9)	
**Probability of food scarcity in future**										
Yes	1,821 (45.3)	369 (20.3)	1,452 (79.7)	<0.001	492 (27)	1,329 (73)	<0.001	654 (35.9)	1,167 (64.1)	<0.001
No	2,199 (54.7)	794 (36.1)	1,405 (63.9)		1,024 (46.6)	1,175 (53.4)		1,085 (49.3)	1,114 (50.7)	

### Regression Analysis

Table 2 shows the results of binary logistic regression analysis by depression, anxiety, and stress. The variables found to be significant in the binary logistic regression analysis were included in the adjusted models. As per as the multivariable logistic analysis (Table 3), females were more likely to have depression, anxiety, and stress compared to males (AOR_*Depression*_: 1.34; 95% CI: 1.15–1.55, *p* < 0.001, AOR_*Anxiety*_: 1.62; 95% CI: 1.41–1.86, *p* < 0.001, and AOR_*Stress*_: 1.79; 95% CI: 1.57–2.05, *p* < 0.001). Similarly, the higher odds of depression, anxiety, and stress were found among participants who reported high family income, poor self-perceived health status, COVID-19 affected people in the local area, food scarcity, and probability of food scarcity in future.

**Table 2:** Binary logistic regression analysis by depression, anxiety, and stress.

**Variables**	**Depression**		**Anxiety**		**Stress**	
	**COR (95% CI)**	***P*** **-value**	**COR (95% CI)**	***P*** **-value**	**COR (95% CI)**	***P*** **-value**
**Age**						
Younger age	Ref.		Ref.		Ref.	
Middle age	0.63 (0.52–0.77)	<0.001	1.10 (0.91–1.34)	0.302	0.62 (0.51–0.75)	<0.001
Older age	0.81 (0.44- 1.47)	0.492	1.43 (0.78–2.64)	0.243	0.65 (0.37–1.15)	0.143
**Sex**						
Male	Ref.		Ref.		Ref.	
Female	1.26 (1.10–1.45)	0.001	1.44 (1.27–1.64)	<0.001	1.72 (1.52–1.95)	<0.001
**Education**						
Below university	1.37 (1.15–1.63)	<0.001	1.61 (1.37–1.90)	<0.001	1.19 (1.02–1.38)	0.027
University	Ref.		Ref.		Ref.	
**Marital status**						
Married	Ref.		Ref.		Ref.	
Unmarried	1.72 (1.41–2.09)	<0.001	0.90 (0.740–1.097)	0.300	1.68 (1.13–2.03)	<0.001
**Family members**						
Up to 4	1.54 (1.17- 2.02)	0.002	1.03 (0.79–1.35)	0.793	1.43 (1.10–1.185)	0.007
5–8	1.17 (0.89- 1.55)	0.255	0.90 (0.69–1.19)	0.691	1.14 (0.88–1.49)	0.304
Above 8	Ref.		Ref.		Ref.	
**Monthly income**						
<20,000 BDT	Ref.		Ref.		Ref.	
20,000–50,000 BDT	1.24 (1.02–1.49)	0.024	1.42 (1.22–1.74)	<0.001	1.40 (1.18–1.67)	<0.001
>50,000 BDT	1.03 (0.85–1.26)	0.713	1.05 (0.88–1.26)	0.555	1.20 (1.00–1.44)	0.043
**Residence**						
Rural	Ref.		Ref.		Ref.	
Urban	1.09 (0.93–1.28)	0.286	1.02 (0.87–1.18)	0.800	1.20 (1.03–1.39)	0.014
**Self-perceived health status**						
Good	Ref.		Ref.		Ref.	
Poor	1.78 (1.31–2.40)	<0.001	2.59 (1.92- 3.48)	<0.001	2.04 (1.57–2.66)	<0.001
**COVID-19 affected people in family**						
Yes	1.21 (0.89–1.64)	0.207	1.93 (1.42–2.62)	<0.001	1.36 (1.03–1.78)	0.026
No	Ref.		Ref.		Ref.	
**COVID-19 affected people in area**						
Yes	1.62 (1.39–1.88)	<0.001	1.58 (1.37-1.82)	<0.001	1.64 (1.43-1.89)	<0.001
No	Ref.		Ref.		Ref.	
**Income status change due to COVID-19**						
Yes	1.78 (1.54–2.05)	<0.001	1.80 (1.58–2.06)	<0.001	1.70 (1.49–1.94)	<0.001
No	Ref.		Ref.		Ref.	
**Possibility of decreasing income**						
Yes	2.24 (1.93–2.59)	<0.001	1.95 (1.69–2.25)	<0.001	1.84 (1.60–2.12)	<0.001
No	Ref.		Ref.		Ref.	
**Probability of losing job**						
Yes	2.27 (1.94–2.65)	<0.001	2.43 (2.11–2.81)	<0.001	1.64 (1.43–1.87)	<0.001
No	Ref.		Ref.		Ref.	
**Food scarcity due to COVID-19**						
Yes	3.18 (2.52- 4.01)	<0.001	4.32 (3.46–5.40)	<0.001	2.01 (1.68–2.40)	<0.001
No	Ref.		Ref.		Ref.	
**Probability of food scarcity in future**						
Yes	2.22 (1.92–2.56)	<0.001	2.35 (2.06–2.68)	<0.001	1.73 (1.53–1.97)	<0.001
No	Ref.		Ref.		Ref.	

**Table 3:** Multivariable logistic regression analysis by depression, anxiety, and stress.

**Variables**	**Depression**		**Anxiety**		**Stress**	
	**AOR (95% CI)**	***P*** **-value**	**AOR (95% CI)**	***P*** **-value**	**AOR (95% CI)**	***P*** **-value**
**Age**						
Younger age	Ref.		–	–	Ref.	
Middle age	0.91 (0.69–1.20)	0.547	–	–	0.88 (0.68–1.14)	0.339
Older age	1.37 (0.69–2.72)	0.355	–	–	1.07 (0.56–2.03)	0.828
**Sex**						
Male	Ref.		Ref.		Ref.	
Female	1.34 (1.15–1.55)	<0.001	1.62 (1.41–1.86)	<0.001	1.79 (1.57–2.05)	<0.001
**Education**						
Below university	0.97 (0.80–1.18)	0.766	1.06 (0.88–1.28)	0.517	0.92 (0.77–0.1.09)	0.357
University	Ref.		Ref.		Ref.	
**Marital status**						
Married	Ref.		–	–	Ref.	
Unmarried	1.87 (1.41–2.49)	<0.001	–	–	1.72 (1.41–2.11)	<0.001
**Family members**						
Up to 4	1.72 (1.28–2.30)	<0.001	–	–	1.53 (1.16–2.02)	0.003
5–8	1.39 (1.04–1.87)	0.026	–	–	1.26 (0.95–1.66)	0.104
Above 8	Ref.		–	–	Ref.	
**Monthly income**						
<20,000 BDT	Ref.		Ref.		Ref.	
20,000–50,000 BDT	1.43 (1.16–1.77)	<0.001	1.70 (1.40–2.06)	<0.001	1.48 (1.22–1.81)	<0.001
>50,000 BDT	1.57 (1.23–2.00)	0.377	1.48 (1.19–1.82)	<0.001	1.56 (1.25–1.94)	<0.001
**Residence**						
Rural	–	–	–	–	Ref.	
Urban	–	–	–	–	0.94 (0.79–1.13)	0.541
**Self-perceived health status**						
Good	Ref.		Ref.		Ref.	
Poor	1.81 (1.32–2.47)	<0.001	2.37 (1.74–3.22)	<0.001	2.12 (1.60–2.79)	<0.001
**COVID-19 affected people in family**						
Yes	–	–	1.39 (1.01–1.93)	0.043	1.13 (0.84–1.51)	0.402
No	–	–	Ref.		Ref.	
**COVID-19 affected people in area**						
Yes	1.28 (1.09–1.50)	0.002	1.24 (1.06–1.44)	0.005	1.31 (1.12–1.53)	<0.001
No	Ref.		Ref.		Ref.	
**Income status change during the COVID-19**						
Yes	1.06 (0.88–1.29)	0.501	1.17 (0.98–1.40)	0.081	1.26 (1.06–1.51)	0.008
No	Ref.		Ref.		Ref.	
**Possibility of decreasing income**						
Yes	1.61 (1.31–1.98)	<0.001	1.18 (0.97–1.44)	0.086	1.37 (1.13–1.67)	0.001
No	Ref.		Ref.		Ref.	
**Probability of losing job**						
Yes	1.38 (1.13–1.67)	0.001	1.37 (1.15–1.64)	<0.001	1.11 (0.94–1.32)	0.209
No	Ref.		Ref.		Ref.	
**Food scarcity during the pandemic**						
Yes	1.97 (1.49–2.60)	<0.001	2.70 (2.07–3.50)	<0.001	1.48 (1.18–1.85)	0.001
No	Ref.		Ref.		Ref.	
**Probability of food scarcity in future**						
Yes	1.40 (1.17–1.69)	<0.001	1.46 (1.23–1.74)	<0.001	1.35 (1.14–1.59)	0.001
No	Ref.		Ref.		Ref.	

Unmarried participants were 2 times more likely to have depression and stress than married participants (AOR_*Depression*_: 1.87; 95% CI: 1.41–2.49, *p* < 0.001, and AOR_*Stress*_: 1.72; 95% CI: 1.41–2.11, *p* < 0.001). Participants who reported smaller families and the possibility of decreasing income in the future had greater odds of depression and stress. In addition, the probability of losing employment was associated with higher odds of depression and anxiety; whereas COVID-19 affected people in the family and income status change during the COVID-19 were associated with anxiety and stress, respectively.

## Discussion

After the severe acute respiratory syndrome (SARS) outbreak in 2003 (44), the COVID-19 pandemic is the largest viral outbreak in modern history, and has had a significant effect on physical and mental health, and the financial status of the general population. Nearly every country in the world has been affected by COVID-19, with many nations undertaking lockdown measures to deter transmission, as a result of which large parts of the population have had to stay at home, and millions have lost their jobs (45). This may affect their financial situation and mental health, and can result in depression, anxiety, stress, frustration, and concerns about the future. It is therefore important to identify which factors are associated with mental health outcomes, so that healthcare and other policies (such as economic, employment, and social policies) can address them.

In the present study, the prevalence estimates of depression, anxiety, and stress were 71.1, 62.3, and 56.7%, respectively. Higher levels of depression, anxiety, and stress were positively associated with female sex, being unmarried, a lower number of family members, higher monthly family income, poor self-perceived health status, the presence of people being infected in their residential area, the probability of decreased income, current food scarcity and the potential for food scarcity in the future, and the possibility of losing employment during the pandemic.

### Comparison With Other Studies

The results of the study were compared to earlier research focused on (i) depression, anxiety and stress [using Patient Health Questionnaire (PHQ-9), Depression, Anxiety, Stress Scale (DASS-21), Generalized Anxiety Disorder (GAD-7), Hospital Anxiety and Depression Scale (HADS), Health Anxiety Inventory (HAI), Geriatric Depression Scale-15], and (ii) studies with a particular focus on financial difficulties during COVID-19.

The results of this study showed that depression, anxiety, and stress were comparatively higher in females than males, in line with other epidemiological research (4, 46), but in disagreement with previous studies in Bangladesh using similar (47) and different instruments (48). It is likely that women may assume additional caretaking duties of their families during the pandemic which may account for their increased mental health problems. In the present study, depression and stress were significantly higher among those who reported being unmarried and with a lower number of family members. Being alone during lockdown can often cause feelings of loneliness and affect mental well-being leading to depression and stress (49, 50), and the present study supports and such findings. A previous study has reported that being married (compared to being single, widowed or separated) can be associated with depression (51), however this utilized data collected prior to the pandemic.

A significant association between high income family (monthly income 20,000–50,000 BDT) and depression, anxiety, and stress emerged in our results, inconsistent with prior Bangladeshi studies (4, 33, 34) which did not find a significant association between income and mental health concerns. More work is needed to ascertain why this is the case. Having family members and other people living their residential area infected with COVID-19 were associated with higher depression, anxiety and stress, consistent with previous studies which have suggested this may be due to fear of infection, enforced quarantine and feeling stigmatized (52). People with poor self-reported health status also exhibited higher odds of depression, anxiety and stress in the present study, consistent with previous Bangladeshi reports (32, 34).

In the present study, financial difficulties due to a reduction in family income, food scarcity, and potential unemployment were found to be statistically significant associated factors of depression, anxiety, and stress. COVID-19 is a highly contagious disease with catastrophic repercussions for humanity and the global economy, as well as for mental health causing psychosocial distress (53). Major victims of the COVID-19 outbreak include micro, small, medium-sized enterprises and also the general population (54). Prolonged country-wide lockdowns and a global economic recession disrupted normal supply chains and created economic hardship (55). A previous study conducted in Bangladesh indicated that the COVID-19 pandemic and partial lockdown had socio-economic impacts including on poorer communities, leading to price increases of basic essentials, disruptions to formal education, and the possibility of a severe socio-economic and health crisis (56). The data we present are a year old as it takes time to analyze data and publish the results. It is possible that these may have changed in the last year and further work may be needed to ascertain any temporal changes.

### Limitations

This study is not without limitations which should be taken into account when interpreting the results. The study was cross-sectional so causality cannot be determined. In this respect, a prospective study will be helpful. Respondents needed to have access to the internet to participate, indicating that they had a higher socio-economic standing than the general population, although research assistants collected data from friends and family members who were not digitally literate. Therefore, the possibility of sampling bias should be considered. Self-reported health status was measured using a binary response and that may have limited the range of responses. Finally, this study relied on self-reported responses regarding experience during home-quarantine stay which may not align with the clinical diagnosis of mental health professionals.

## Conclusions

The present study demonstrates that mental health disturbances are prevalent among residents in Bangladesh, and financial difficulties correlate with mental health issues. Psycho-social support needs to be strengthened for people living in Bangladesh. The government, non-governmental agencies and other workplaces should support employees during the pandemic and in its aftermath rather than cutting jobs or salaries. The findings should inform mental health strategies to increase mental resilience during the COVID-19 pandemic in Bangladesh. Furthermore, the present study will contribute to future research in Bangladesh.

## Data Availability Statement

The raw and clean data files were served as Supplementary Material.

## Ethics Statement

The studies involving human participants were reviewed and approved by Institutional Review Board of the Biological Science Faculty, Jessore University of Science & Technology, Jessore-7408, Bangladesh [Ref: ERC/FBS/JUST/2020-43(a)]. The participants provided their written informed consent to participate in this study.

## Author Contributions

AS, MI, SN, and ZS: conceptualization. AS, MI, SN, and TK: methodology. AS and MI: data curation, writing—original draft preparation, formal analysis, and investigation. MI, SP, and KK: writing—review and editing. MZA, MIA, SN, TK, and ZS: resources. MZA and KK: supervision. All authors read and approved the final manuscript.

## Conflict of Interest

The authors declare that the research was conducted in the absence of any commercial or financial relationships that could be construed as a potential conflict of interest.

## Publisher's Note

All claims expressed in this article are solely those of the authors and do not necessarily represent those of their affiliated organizations, or those of the publisher, the editors and the reviewers. Any product that may be evaluated in this article, or claim that may be made by its manufacturer, is not guaranteed or endorsed by the publisher.
